# Could the Immune Modulation of Invariant Natural Killer T Cell and the Expression of CD244 and CD196 on Natural Killer Cell Subtypes Impact the Treatment Outcomes of Acute Myeloid Leukemia Patients?

**DOI:** 10.14740/jh2209

**Published:** 2026-08-31

**Authors:** Asmaa M. Zahran, Amal Rayan, Ahmed Refaat, Ahmad Khalid Ibrahim Elsayh, Nour Y.H. Hussein, Marwa A. Sabet, Salwa Seif Eldin, Aya Fergany, Merna W. Narouz, Yomna R. Mahboob, Zeinab Albadry M. Zahran

**Affiliations:** a Clinical Pathology Department, South Egypt Cancer Institute, Assiut University, Assiut, Egypt; b Clinical Oncology Department, Faculty of Medicine, Assiut University, Assiut, Egypt; c Department of Medical Oncology, South Egypt Cancer Institute, Assiut University, Assiut, Egypt; d Faculty of Medicine, Assiut National University, Assiut, Egypt; e Department of Microbiology and Immunology, Faculty of Pharmacy, Sphinx University, New Assiut, Egypt; f Department of Medical Microbiology and Immunology, Faculty of Medicine, Assiut University, Assiut, Egypt; g Princess Nourah bint Abdulrahman University, Riyadh, Saudi Arabia; h Microbiology and Immunology Department, Faculty of Pharmacy, New Valley University, Egypt; i Department of Internal Medicine, Clinical Hematology Unit, Faculty of Medicine, Assiut University, Assiut, Egypt; j Clinical Pathology Department, Faculty of Medicine, Assiut University, Assiut, Egypt

**Keywords:** Natural killer cells, Invariant natural killer T cells, CD244, CD196, AML, SDG3

## Abstract

**Background:**

The present study aimed to assess the CCR6 and CD244 expression on natural killer (NK) and natural killer T (NKT) cells, considering their different subsets and examining their associations with molecular cytogenetics and clinical outcomes in newly diagnosed adult acute myeloid leukemia (AML) patients.

**Methods:**

We recruited 26 new cases of newly diagnosed adult AML and 24 adult healthy participants as controls, after diagnosis of AML, bone marrow studies, cytogenetics, and molecular studies; samples of peripheral blood were collected from all participants for flow cytometry to determine the expressions of CD244 and CD196.

**Results:**

Invariant NKT cells were significantly more abundant in controls than in patients. In patients, CD244 expression was associated with increased accumulation of NKT cells, whereas CD196^+^ NKT cells were more frequently found in controls. Regarding CD3^−^ NK cells, co-expression of CD244 and CD196 was significantly increased in patients compared with controls across all CD3^−^ NK subpopulations. CD244 expression was higher in controls, within CD16^bright^CD56^bright^ NK, CD16^bright^CD56^dim^ NK, and CD16^dim^CD56^bright^ NK subsets. In contrast, in patients’ CD16^+^CD56^−^ NK cells, CD196 showed an inverse pattern: it was expressed more in patients with CD16^bright^CD56^dim^ NK cells, compared with controls’ CD16^+^CD56^−^ and CD16^−^CD56^+^ NK cells. Dim CD56 expression on NK cells, regardless of CD16 level, was associated with treatment remission. In addition, the presence of CD244 and/or CD196 was linked to treatment failure, whereas the dual absence of CD244 and CD196 was associated with remission.

**Conclusion:**

The expression pattern of CD244 (2B4 receptor) and CD196 on NK and NKT cells was generally associated with remission status and prognostic features of AML patients.

## Introduction

In adults, acute myeloid leukemia (AML) is the most common type of acute leukemias representing about 80% of all leukemias, but still fairly rare to account for 1% of all cancers with an average age at diagnosis of 68 years. Although it is slightly more common in males than females, the average lifetime risk of having AML in both sexes is 0.5–1% [1], and only about 15% of patients older than 60 years remain alive at 5 years and increase up to 40% for patients younger than 60 years [2]. Treatment of AML remains largely unchanged over years with multiagent chemotherapy being the mainstay therapy.

Tumor microenvironment (TME) of hematologic malignancies differs considerably from that of solid ones. For leukemias, bone marrow (BM) harbors most leukemic blasts, while secondary lymphoid organs such as lymph nodes and spleen constitute components of TME. Being well described in several hematologic malignancies including Hodgkin’s disease (HD), chronic myeloid leukemia (CML), acute lymphoblastic leukemia (ALL), and chronic lymphocytic leukemia (CLL), the immune microenvironment is still less defined in AML [3]. Among TME cells are natural killer (NK) cells which are a group of innate cells showing spontaneous cytolytic activity against stressed cells like tumor cells, and are phenotypically identified by expression of CD56 (neural cell adhesion molecule), CD16 (human IgG Fc receptor III, FcγRIII), and lack of CD3, T-cell receptors, and CD19 [4]. NK cells modulate the functions of other innate and adaptive immune cells mediated by their secretions of cytokines such as interferon-γ (IFN-γ), tumor necrosis factor-α, granulocyte monocyte colony-stimulating factor, and chemokines. Furthermore, they represent 5–15% of human peripheral blood (PB) mononuclear cells, and are found in many tissues in addition to BM and PB, including kidney, gut, liver, joints, breast, thymus, and uterus [5].

Natural killer T (NKT) cells are immune cells harboring the characteristics of both innate and adaptive immune cells. NKT cells express a T-cell receptor (TCR), developed because of somatic DNA rearrangement. However, whereas the TCR repertoire of conventional T cells is highly diverse, most NKT cells, are commonly referred to as invariant or type I NKT cells [6].

Invariant NKT (iNKT) cells, a population of mature T cells, are identified by lack of expression of CD4 or CD8 or both (double negative (DN)) but expressing TCR and NK cell marker NK1.1. They required CD1d expression for their development and recognition by lipids instead of peptides [7]. These cells are known as type I NKT cells to represent about 0.01–0.1% of T-cell population in human [8]. DN and CD8^+^ iNKT cells are associated with increased IFN-γ secretion and cytotoxic functions, while CD4^+^ iNKT cells produce Th2 type cytokines such as interleukin (IL)-4 and IL-13 [9]. Furthermore, human iNKT cells express NK-related markers such as CD244, CD94, CCR6, NKG2A, and NKG2D. iNKT cells were found to be enriched in the adipose tissue and omentum of human compared to the liver in mice and their frequency in PB was much lower than mice [10].

CD244 receptor (2B4 receptor), an Ig superfamily signaling lymphocyte activation molecule (SLAM) family receptor, binds CD48 which is a transmembrane receptor ubiquitously expressed on hematopoietic cells, is expressed on all NK cells [11], dendritic cells (DCs), T cells, and myeloid derived suppressor cells (MDSCs), and acts as inhibitory or activating receptor based on the adaptor protein recruited at the cytoplasmic tail. In human, CD244 has mainly an activating function that can be disturbed by the relative expression of SAP, EAT-2, and ERT molecules concerned in the downstream signaling [12]. CD244–CD48 interaction is essential for optimal proliferation of NK cells in response to IL-2 and cytokine secretion [13]. In addition, the inhibitory CD244 signaling in NK cells is demonstrated in TME and mediated by CD48^+^CD68^+^ macrophages, resulting in early increased INF-γ and TNF-α followed by exhaustion with decreased cytokine production and NK cell apoptosis [14]. TME enhanced the inhibitory function of CD244 through increasing its density and decreasing concentration of functional SAP [15].

CD196, chemokine receptor 6 (CCR6), is a protein belonging to a family A of G-coupled receptor superfamily, expressed on B cells, immature DC, NK cells, NKT cells, T cells, and neutrophils [16]. It also binds chemokine ligand CCL20. Generally, chemokine receptors are comprised of seven transmembrane proteins which are classified into four major groups of CXCR, CCR, CX3CR, and XCR to which four groups of chemokines are bound (CXCL, CCL, CL, and CX3CL) and are included in cellular development, differentiation, tissue distribution, and functions [17, 18]. NK cells express CXCR1, CXCR3^+^, and CXCR4^+^ and contain subsets expressing CCR1, CCR4, CCR5, CCR6, CCR9, CXCR5, and CXCR6. These chemokine receptors are expressed mainly by CD56^high^ NK cells [19, 20].

The present study aimed to assess the CCR6 and CD244 expression on NK and NKT cells, considering their different subsets and examining their associations with molecular cytogenetics and clinical outcomes in newly diagnosed adult AML patients.

## Materials and Methods

The study was conducted in South Egypt Cancer Institute and Assiut University Hospital and involved *de novo* AML cases. We recruited 26 new cases and 24 adult healthy participants as controls from January 2022 to December 2023. The study was approved by Ethical Committee of Faculty of Medicine, Assiut University (IRB 17300648), all methodology were carried out in compliance with Helsinki Declaration, and according to Sustainable Development Goals SDG3 of Assiut University. Written informed consent was gathered from all participants after describing the nature and aim of the current study assuring that participants’ confidentiality was protected. Participation was entirely voluntary, and they were able to withdraw at any time without providing any reason and their data were destroyed if they wish.

It was a prospective case–control study and carried out to determine the expressions of CD244 and CD196 on CD3^+^CD56^+^CD16^+^ NKT cells to characterize four subpopulations of NKT cells: CD3^+^CD56^+^CD16^+^CD196^+^CD244^+^, CD3^+^CD56^+^CD16^+^CD244^−^CD196^−^, CD3^+^CD56^+^CD16^+^CD244^−^CD196^+^, and CD3^+^CD56^+^CD16^+^CD244^+^CD196^−^ cells in PB of AML cases and healthy individuals. Furthermore, the study aimed to determine the subtypes of CD3^−^CD56^+^CD16^+^ NK cells: CD3^−^CD56^bright^CD16^bright^, CD3^−^CD56^bright^CD16^dim^, CD3^−^CD56^dim^CD16^bright^, CD3^−^CD56^dim^CD16^dim^ NK cells and, CD3^−^CD56^+^CD16^−^, CD3^−^CD56^−^CD16^+^ cells, and also to evaluate the expression of CD244 and CD196 on these cells.

Patients were diagnosed based on clinical data developed as a result of progressive cytopenia, as well as oncologic emergencies including renal failure secondary to tumor lysis and hyperuricemia, neurologic and respiratory distress secondary to leukostasis, disseminated intravascular coagulation (DIC), and central nervous system (CNS) involvement [21, 22]. The presence of 20% blasts in PB or BM is the hallmark of AML diagnosis, in addition to flow cytometric immunophenotyping and cytogenetics studies [23].

### Induction chemotherapy

This is the standard of care for AML treatment in younger patients, elderly with low risk of treatment-related mortality, and those with favorable and intermediate risk factors. It consisted of 7 + 3 regimen with 7 days of continuous infusion of cytarabine along with 1–3 days of anthracycline infusion. Higher doses of cytarabine or the addition of fludarabine or etoposide to cytarabine and idarubicin regimen were required in refractory cases and patients without complete response as a reinduction regimen, and elderly patients with high risk of treatment-related mortality received decitabine or etoposide.

Based on the etiology, patients were classified into primary AML and secondary AML on top of myelodysplastic syndrome (MDS). Patients with t(8;21), inversion of chromosome 16, and genetic mutations in *NPM1* gene were considered to have favorable prognosis, those with *FLT3* gene abnormalities, del 5q, del 9q, t(9:22), monosomy 5, and monosomy 7 had unfavorable prognosis, and those with trisomy 6, 8, and del Y had intermediate prognosis.

Patients with blast counts less than 5% in their BM after induction chemotherapy were considered to have complete remission and shifted to consolidation therapy and bone marrow transplantation (BMT).

### Flow cytometric detection of the expression of CD196 and CD244 on NKT cells and NK cell subtypes

**Figure 1:**
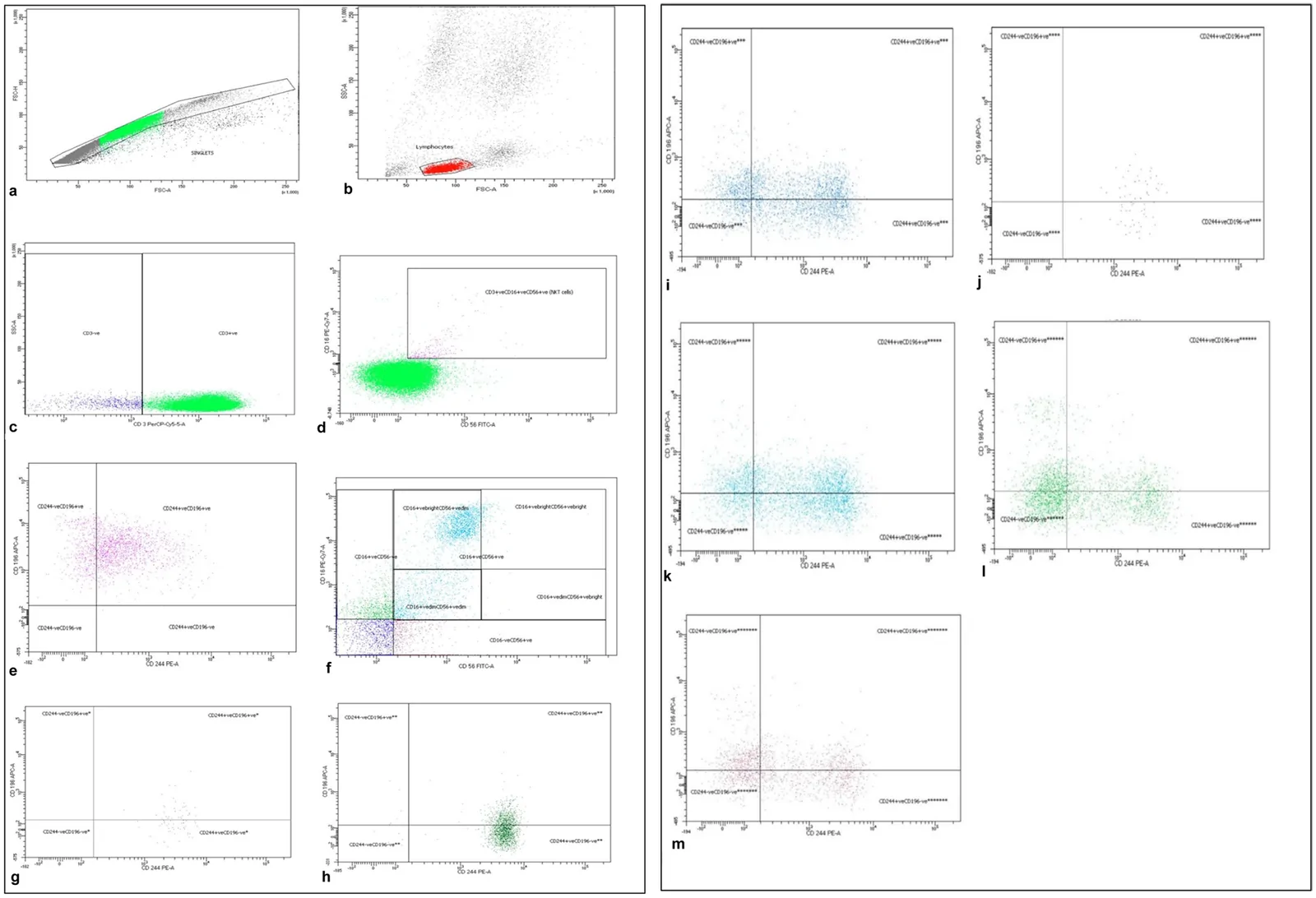
Flow cytometric detection of lymphocytes, NK cells, NKT cells, and their expression of CD196 and CD244. (a) Forward scatter area and height to define singlets and exclude duplicates. (b) Forward and side scatter dot plot was used to define lymphocyte population. (c) The expression of CD3 on lymphocyte population to detect CD3^+^ and CD3^−^ lymphocytes. (d) The expression of CD16 and CD56 on CD3^+^ lymphocyte population to detect CD3^+^CD16^+^CD56^+^ NKT cells. (e) The expression of CD196 and CD244 on CD3^+^CD16^+^CD56^+^ NKT cells. (f) The expression of CD16 and CD56 on CD3^−^ population to detect different NK cell subtypes according to the positivity or negativity, dimness, or brightness of CD16 and CD56. (g) The expression of CD196 and CD244 on CD3^−^CD16^+bright^CD56^+bright^ NK cells. (h) The expression of CD196 and CD244 on CD3^−^CD16^+bright^CD56^+dim^ NK cells. (i) The expression of CD196 and CD244 on CD3^−^CD16^+dim^CD56^+dim^ NK cells. (j) The expression of CD196 and CD244 on CD3^−^CD16^+dim^CD56^+bright^ NK cells. (k) The expression of CD196 and CD244 on total CD3^−^CD16^+^CD56^+^ NK cells. (l) The expression of CD196 and CD244 on CD3^−^CD16^+^CD56^−^ population. (m) The expression of CD196 and CD244 on CD3^−^CD16^−^CD56^+^ population.

The PB was stained for 20 min with CD3-V450, CD56-FITC, CD196-APC, CD16-PE-CY7, and CD244-PE (Becton Dickinson Biosciences, CA, USA). After red blood cell (RBC) lysis and washing, the cells were resuspended in phosphate-buffered saline (PBS), and analyzed by FACSC antomated flow cytometry (BD Biosciences, USA). Forward and side scatter histogram was used to define the lymphocytes population. The expression of CD3 was assessed on the lymphocytes, and then, the CD3^+^ and CD3^−^ were gated for further analysis. Then the expression of CD16 and CD56 on CD3^−^ cells was assessed to detect NK cells. The expressions of CD56 and CD16 were assessed on CD3^+^ lymphocytes to detect NKT cells. NK cells were then divided into different NK cell subtypes according to the positivity or negativity, dimness, or brightness of CD16 and CD56. Then, the expression of CD196 and CD244 on NKT cells and NK cell subtypes was assessed. The results are expressed as percentages and shown in Figure 1.

### Statistical analysis

Data were analyzed using IBM-SPSS version 27 and GraphPad prism version 8.4.0 and considered significant at P-value of < 0.05. All immune cells were not normally distributed with Shapiro–Wilk test (P < 0.05). Descriptive statistics were expressed as mean ± standard deviation (SD), range, median, and percentages, while inferential statistics were analyzed by Mann–Whitney test, Robust test, and Fisher’s exact. All were carried out for significance.

## Results

The current results revealed comparability of median age and sex distribution between patients and healthy volunteers with median age of 46 years and male predominance in patients as mentioned in Table 1.

**Table 1:** Demographic Data of Study Population

Demographic	Patients (n = 26)	Controls (n = 24)	P-value
Age^a^			0.6
Mean ± SD	45.5 ± 18.1	47.6 ± 6.7	
Median	46 years	47 years	
Sex^b^			0.7
Male (n = 31)	17 (54.8%)	14 (45.2%)	
Female (n = 19)	9 (47.4%)	10 (52.6%)	

Data are expressed as mean ± SD, median, number, and percentages. ^a^Mann–Whitney test. ^b^Fisher’s exact test. P ≤ 0.05 is considered significant. SD: standard deviation.

BM suppression with anemia, thrombocytopenia, leukocytosis with subsequent hyperuricemia, increased lactate dehydrogenase (LDH), serum ferritin, and C-reactive protein, decreased fibrinogen level in six patients with increased risk of DIC (four patients) and increased D-dimer (five patients) were the predominant laboratory findings (Table 2).

**Table 2:** Laboratory Characteristics of AML Patients

Characteristics	Descriptive
Hb (mean ± SD)	8.1 ± 1.4
WBCs (mean ± SD)	74.9 ± 77.5
Lymphocytic count (mean ± SD)	15.9 ± 6.1
Neutrophilic count (mean ± SD)	24.3 ± 13.1
Platelet count (mean ± SD)	48.14 ± 38.8
LDH (mean ± SD)	929.3 ± 735.6
Uric acid (mean ± SD)	9.6 ± 4.8
Serum ferritin (mean ± SD)	606.1 ± 643.04
CRP (mean ± SD)	46.4 ± 54.8
Peripheral blasts % (mean ± SD)	52.9 ± 19.5
BM blasts % (mean ± SD)	57.7 ± 19.8
Impaired liver function	10 (38.5%)
Raised renal function	6 (23.1%)
DIC, %	4 (15.4%)
Decreased fibrinogen level, %	6 (23.1%)
Raised D-dimer, %	5 (19.2%)
Impaired coagulation, %	5 (19.2%)

Data are expressed as mean ± SD, numbers, and percentages. AML: acute myelocytic leukemia; CRP: C-reactive protein; DIC: disseminated intravascular coagulation; Hb: hemoglobin; LDH: lactate dehydrogenase; SD: standard deviation; WBC: white blood cell.

Only three cases were found to have secondary AML on top of MDS. More than half of cases were M4/M5, and unfavorable cytogenetics, and the common molecular genetic abnormalities were NPM1, FLT3 mutations, and CD117 expression (Table 3).

**Table 3:** AML Types, Cytogenetics, Molecular Genetics, and Risk Groups

Characteristics	Frequencies
AML type	
Primary	23 (88.5%)
Secondary	3 (11.5%)
FAB types	
M1	2 (7.7%)
M2	1 (3.8%)
M1/M2	4 (15.4%)
M4	2 (7.7%)
M4/M5	14 (53.8%)
M5	3 (11.5%)
Cytogenetics	
Favorable	12 (46.2%)
Unfavorable	14 (53.8%)
Molecular genetics	
Positive FLT3	9 (34.6%)
Positive CD117	8 (30.8%)
Positive NPM1	8 (30.8%)

Data are expressed as numbers and percentages. AML: acute myelocytic leukemia; FAB: French-American-British.

### Differential expression of NKT subpopulations including iNKT between AML patients and controls

**Figure 2:**
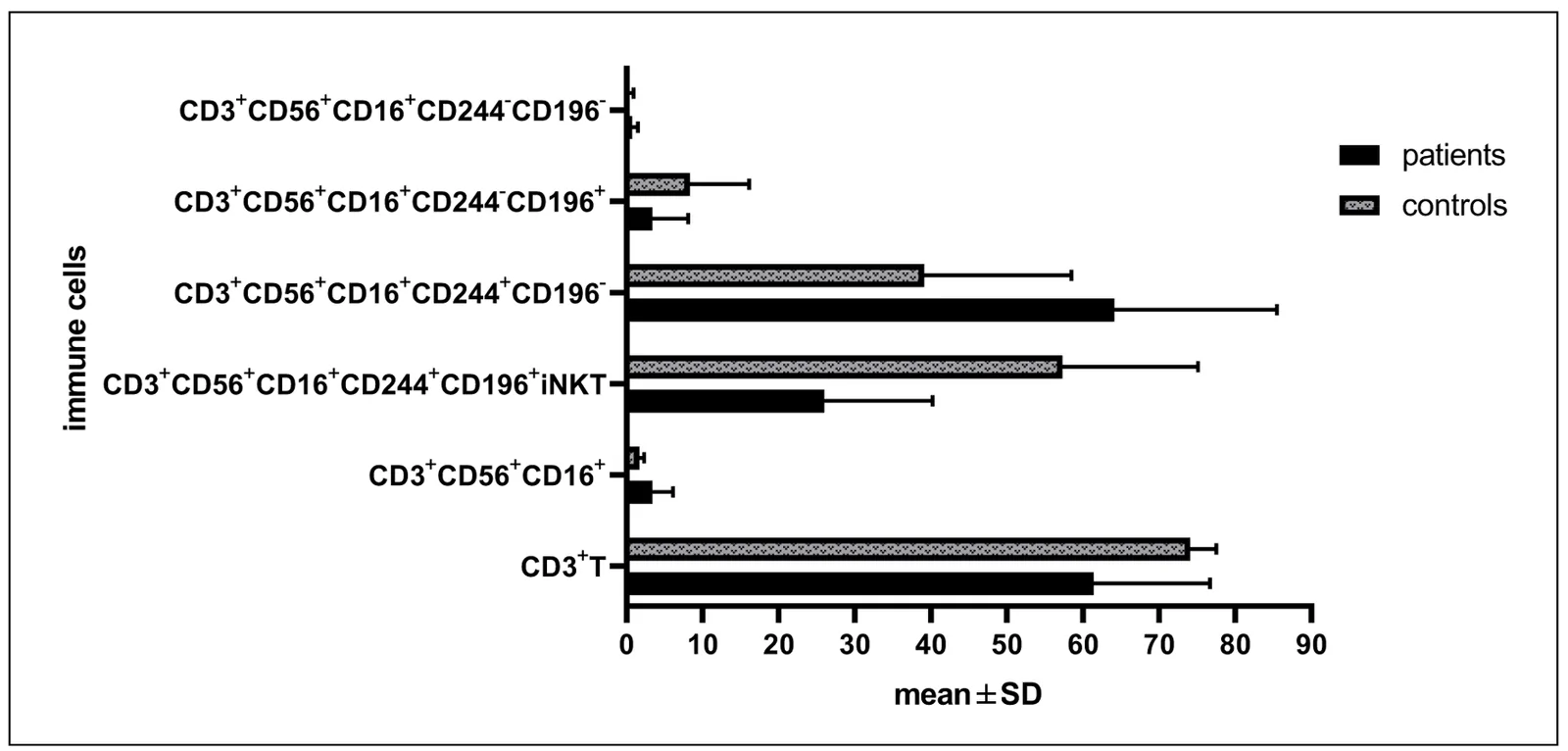
Differential expressions of CD244 and CD196 on CD3^+^CD56^+^CD16^+^ cells between AML patients and healthy controls, Mann–Whitney test.

The total CD3^+^ T lymphocytic mean percentage was significantly higher in controls compared with patients; however, NKT cells were significantly expressed in patients, iNKT subgroup behaved in a different way, and they were significantly expressed in controls compared with patients. The fraction of NKT cells expressing CD244 was higher in patients while that expressing CD196 was higher in controls with a significant impact. The mean percentage of NKT cells losing the expression of CD244 and CD196 was insignificantly higher in patients compared with controls (Supplementary Material 1, jh.elmerpub.com; Fig. 2).

**Figure 3:**
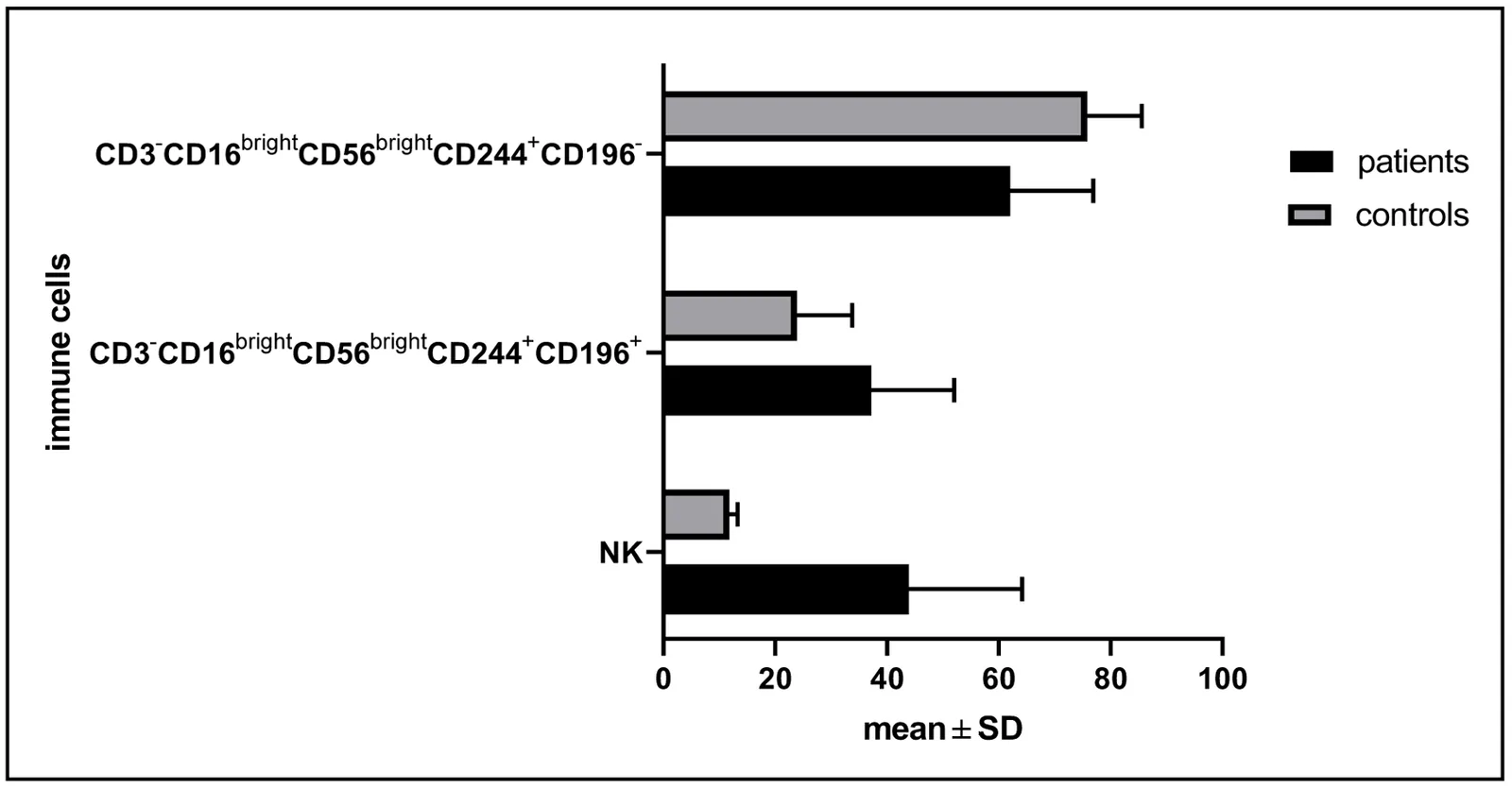
Differential expression of CD244 and CD196 on CD56^bright^CDbright cells between patients and controls.

We detected significant accumulation of NK cells in patients compared with controls (43.9 ± 20.3 vs. 11.8 ± 1.5, P < 0.001). Regarding CD3^−^CD16^bright^CD56^bright^ subpopulation, we explored significant accumulations of those with co-expression of CD244 and CD196 in patients compared with controls; conversely, the subpopulation losing CD196 expression was accumulated in controls. Moreover, we revealed no significant differences in the total CD3^−^CD16^bright^CD56^bright^, the subpopulations losing CD244 and both CD244 and CD196 between patients and controls (Supplementary Material 2, jh.elmerpub.com; Fig. 3).

### Differential expression of CD244 and CD196 on CD16^bright^CD^dim^ subpopulation

**Figure 4:**
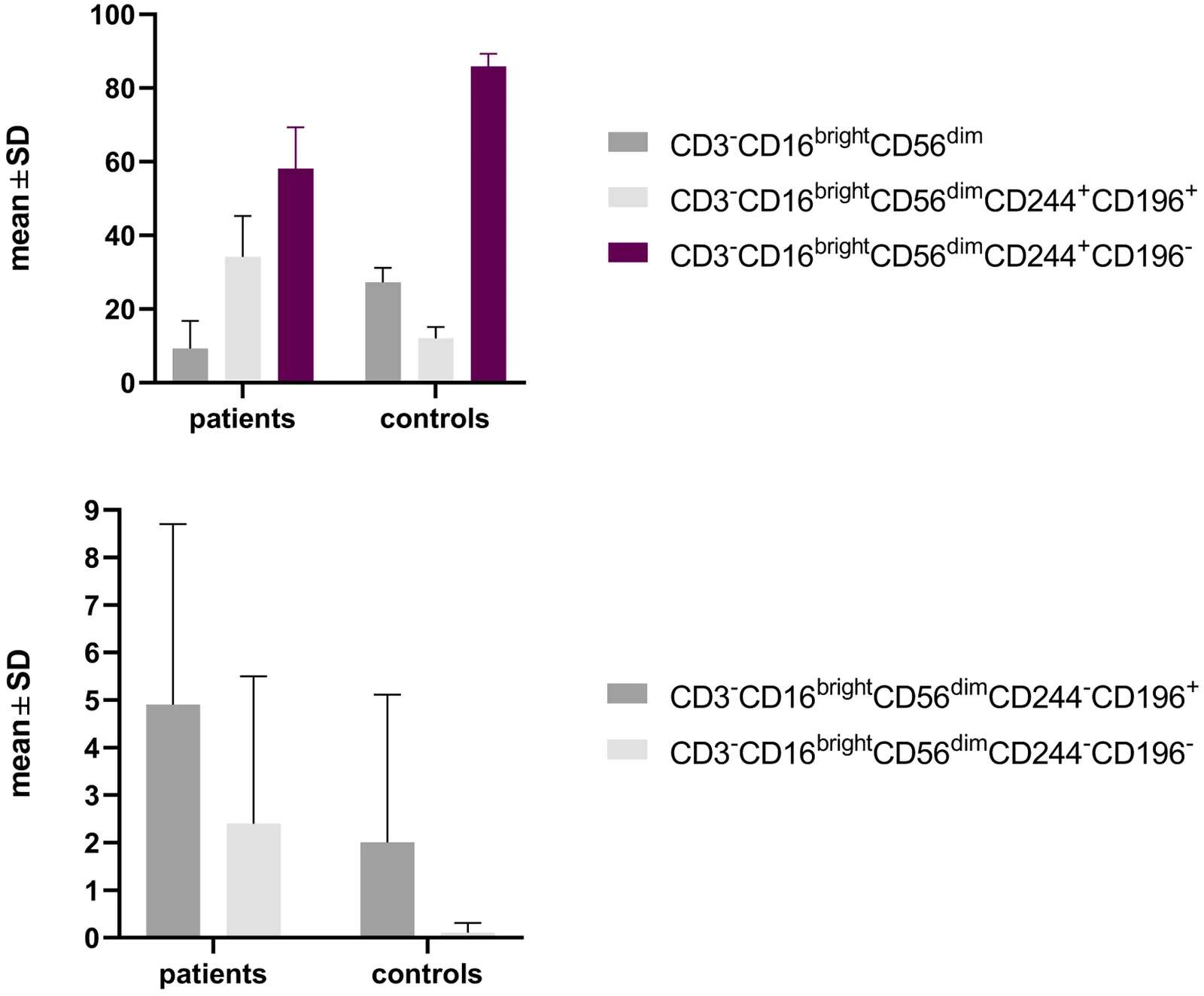
Differential expression of CD16^bright^CD56^dim^ between patients and controls with CD244 and CD196 expression.

The total CD3^−^CD16^bright^CD56^dim^ NK cells and those cells expressing only CD244 were significantly accumulated in controls compared with patients; however, the populations co-expressing both CD196 and CD244, those without co-expression of both CD196 and CD244, and the population with only CD196 expression were significantly increased in patients compared with controls (Supplementary Material 3, jh.elmerpub.com; Fig. 4).

### Differential expression of CD244 and CD196 on CD3^−^CD16^dim^CD56^bright^ NK subpopulation

Regarding CD3^−^CD16^dim^CD56^bright^ NK cells, we detected a significant increase in the population expressing only CD244 in controls compared with patients; conversely, those subpopulations co-expressing CD244 and CD196 markers were increased in patients compared with controls (Table 4; Supplementary Material 4, jh.elmerpub.com).

**Table 4:** Differential Expression of CD244 and CD196 on CD3^−^CD16^dim^CD56^bright^ NK Cells Between Patients and Controls

CD3^−^CD16^dim^CD56^bright^ subpopulations	Group	Mean% ± SD	P-value
CD3^−^CD16^dim^CD56^bright^	Patients	1.1 ± 2.1	0.6
	Controls	0.9 ± 0.4	
CD3^−^CD16^dim^CD56^bright^CD244^+^CD196^+^	Patients	26.7 ± 25.2	0.016
	Controls	12.2 ± 11.3	
CD3^−^CD16^dim^CD56^bright^CD244^+^CD196^−^	Patients	38.4 ± 28.8	< 0.001
	Controls	80.3 ± 8.2	
CD3^−^CD16^dim^CD56^bright^CD244^−^CD196^−^	Patients	0.09 ± 0.4	0.4
	Controls	0.01 ± 0.0	
CD3^−^CD16^dim^CD56^bright^CD244^−^CD196^+^	Patients	0.4 ± 1.3	0.9
	Controls	0.3 ± 1.1	

Data are expressed as mean % ± SD and analyzed by Mann–Whitney test. P ≤ 0.05 is significant.

Our results indicated that the total CD3^−^CD16^dim^CD56^dim^ NK subpopulation with those co-expressing of CD244 and CD196 or lost expressions of both markers was significantly accumulated in patients compared with controls (Table 5; Supplementary Material 4, jh.elmerpub.com).

**Table 5:** Differential Expression of CD244 and CD196 on CD3^−^CD16^dim^CD56^dim^ NK Cells Between Patients and Controls

CD3^−^CD16^dim^CD56^dim^ subpopulations	Group	Mean % ± SD	P-value
CD3^−^CD16^dim^CD56^dim^	Patients	22.02 ± 13.8	< 0.001
	Controls	5.7 ± 2.6	
CD3^−^CD16^dim^CD56^dim^CD244^+^CD196^+^	Patients	33.1 ± 11.6	< 0.001
	Controls	18.4 ± 7.2	
CD3^−^CD16^dim^CD56^dim^CD244^+^CD196^−^	Patients	46.6 ± 19.0	0.3
	Controls	54.4 ± 18.2	
CD3^−^CD16^dim^CD56^dim^CD244^−^CD196^+^	Patients	12.6 ± 11.6	0.2
	Controls	16.0 ± 3.7	
CD3^−^CD16^dim^CD56^dim^CD244^−^CD196^−^	Patients	7.7 ± 7.2	< 0.001
	Controls	1.5 ± 1.5	

Data are expressed as mean % ± SD and analyzed by Mann–Whitney test. P ≤ 0.05 is significant.

### NK subpopulations with lost expressions of either CD16 or CD56

**Figure 5:**
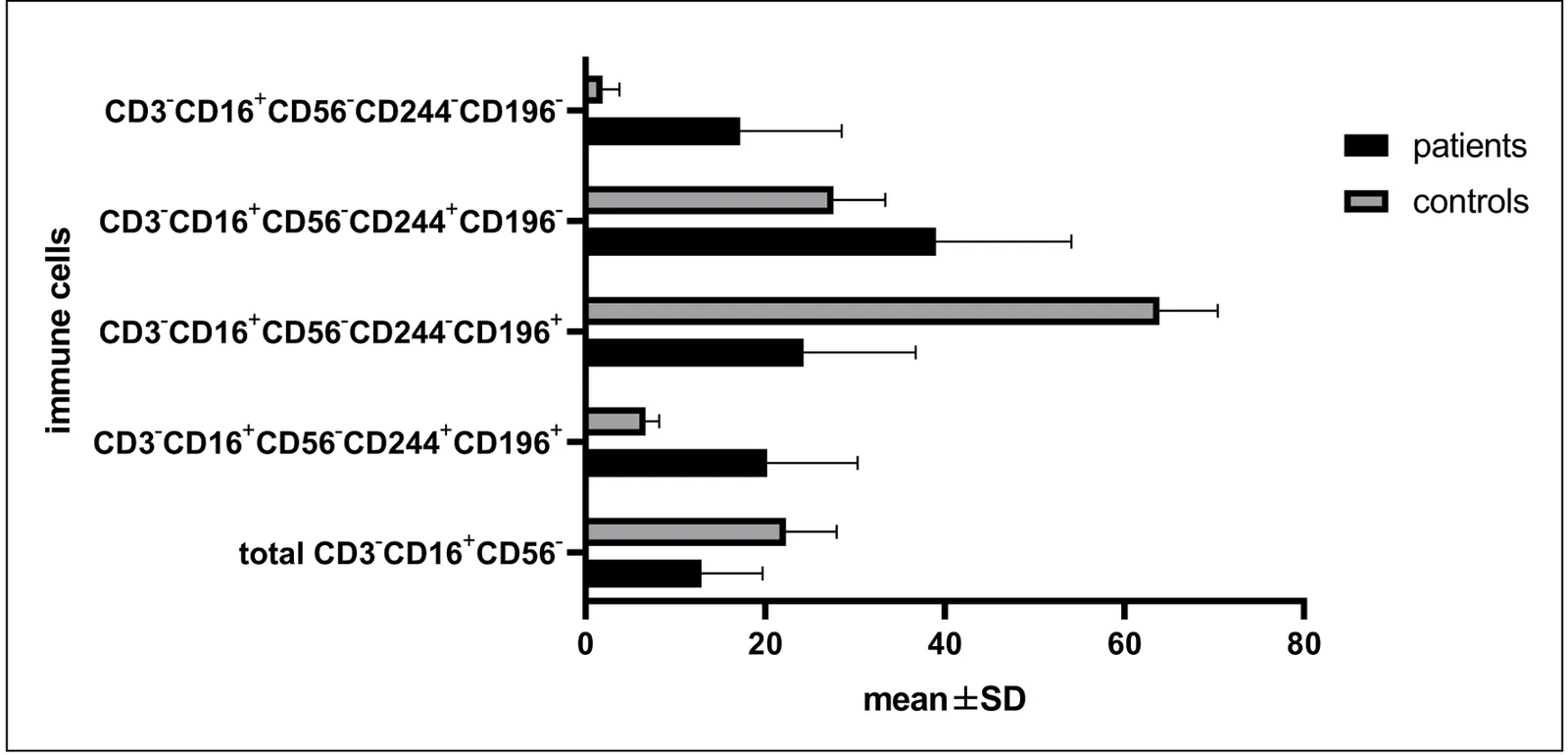
Differential expression of CD3^−^CD16^+^CD56^−^ subpopulations between patients and controls.

Regarding CD3^−^CD16^+^CD56^−^ NK cells, the subpopulations with co-expression, co-absence of CD244 and CD196, and those with lost expression of CD196 only were significantly accumulated in patients compared with controls, while the total population and the fraction of them losing the expression of CD244 were significantly increased in controls (Supplementary Material 5, jh.elmerpub.com; Fig. 5).

**Figure 6:**
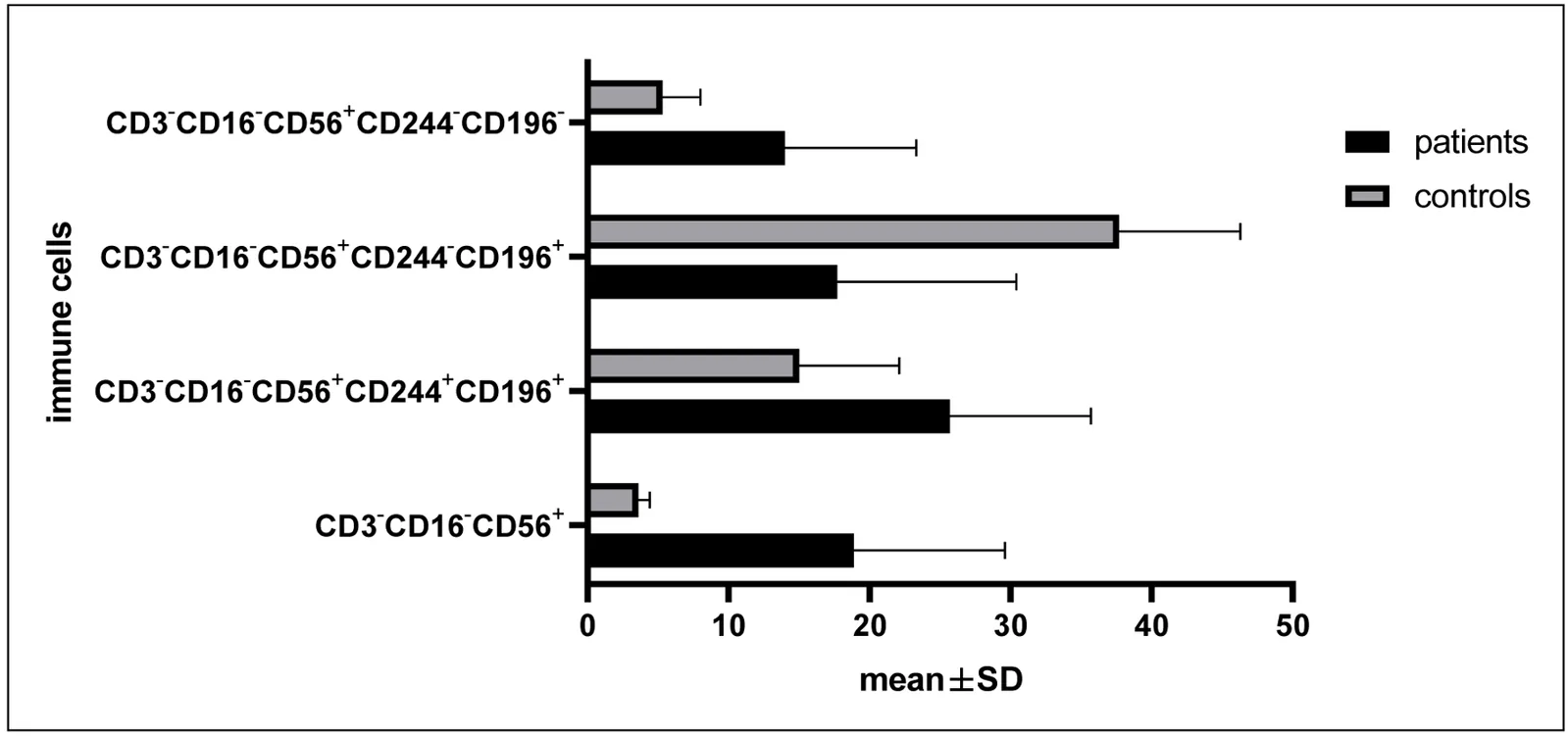
Differential expression of CD3^−^CD16^−^CD56^+^ subpopulations between patients and controls.

Regarding CD3^−^CD16^−^CD56^+^ cells, the mean percentage, and the subpopulations co-expressing and losing both CD244 and CD196 were accumulated in patients compared with controls, while those losing only the expression of CD244 were accumulated in the controls compared with patients (Supplementary Material 5, jh.elmerpub.com; Fig. 6).

Seventeen patients (65%) achieved complete remission, with the most commonly used chemotherapy regimen being 3+7 regimen in > 61% of patients followed by low-dose Ara C (Table 6).

**Table 6:** Type of Chemotherapy Received and Responses

Type	Descriptive
Chemotherapy	
3+7 regimen	16 (61.5%)
Low-dose Ara C	9 (34.6%)
ATRA + low-dose Ara C	1 (3.8%)
Response	
Remission	17 (65.4%)
No remission	9 (34.6%)

Data are expressed as numbers and percentages. Ara C: cytarabine; ATRA: all-trans retinoic acid.

### Relation of NKT cells to remission

**Figure 7:**
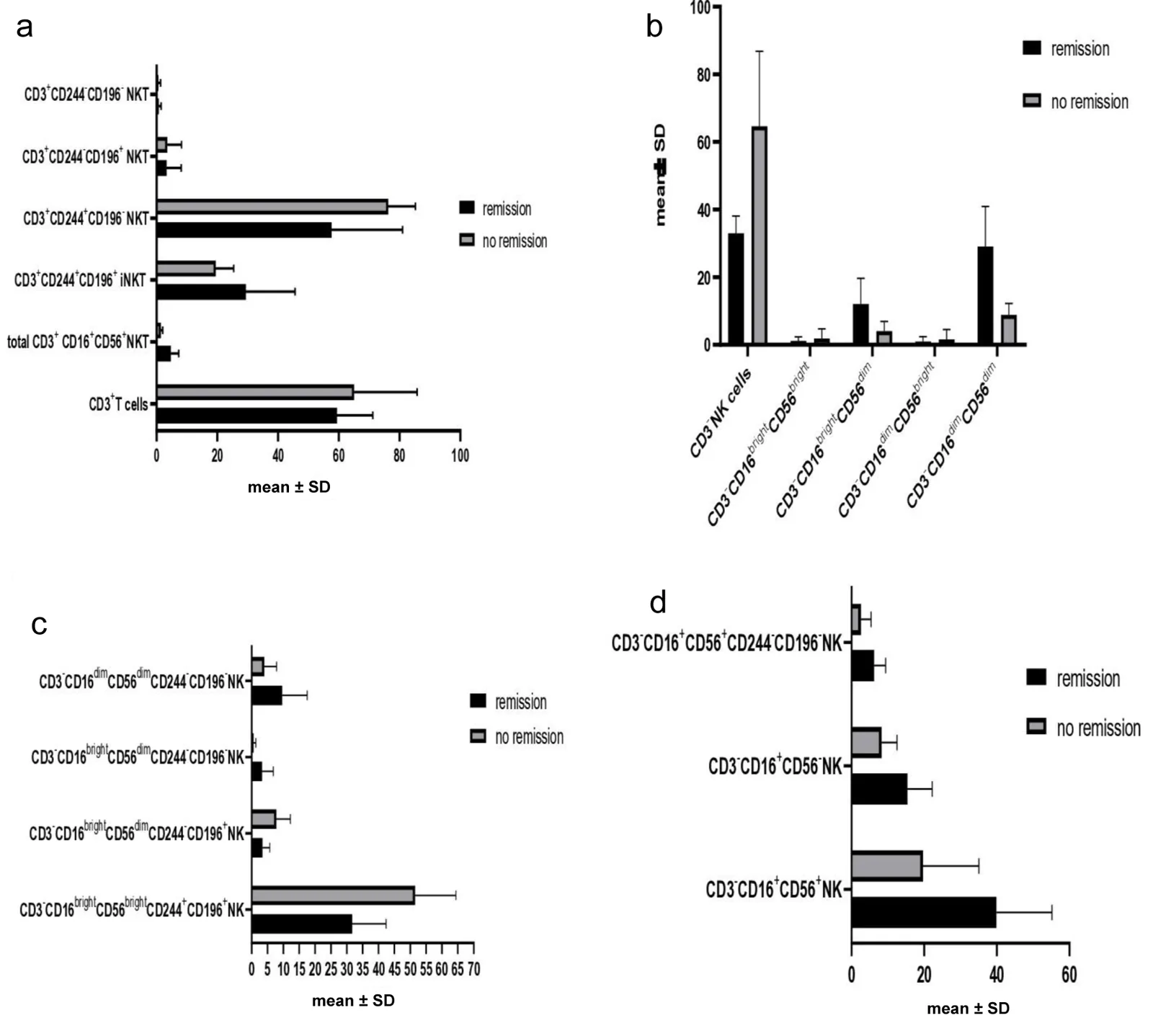
(a) Differential expression of NKT subpopulations according to remission. (b) Differential expression of NK subpopulations. (c) Differential expression of CD244 and CD196 on NK subpopulations. (d) Differential expression of CD16 and CD56 on NK cells according to remission.

The current results revealed significant accumulations of the mean percentage of CD3^+^CD16^+^CD56^+^ NKT cells, and those expressing both CD244 and CD196 (iNKT cells) in patients who achieved remission compared with no remission (4.7 ± 2.6 vs. 1.4 ± 0.6, P < 0.001 and 29.4 ± 16.2 vs. 19.5 ± 5.4, P = 0.034, respectively); however, NKT cells that lost the expression of CD196 were significantly accumulated in those without remission compared to remission (76.3 ± 8.9 vs. 57.6 ± 23.4, P = 0.008), while no significant differences were observed in mean percentage of CD3^+^ T cells and NKT cells that lost the expression of CD244 with or without CD196 according to remission status (59.4 ± 11.8 vs. 65.0 ± 20.8, P = 0.4, 3.3 ± 2.8 vs. 3.5 ± 4.7, P = 0.5, and 0.7 ± 0.8 vs. 0.6 ± 0.7, P = 0.4, respectively for remission compared with no remission) (Fig. 7a).

### Relation of NK with the expressions of CD16 and CD56 subpopulations to response

The mean percentage of NK cells was significantly accumulated in those without remission compared to those with remission (64.6 ± 22.2 vs. 33.0 ± 5.1, P = 0.003). NK cells with low expressions of CD56 having whether bright or dim expression of CD16 were significantly higher in remitting patients compared to those without remission (12.04 ± 7.7 vs. 4.02 ± 2.9, P < 0.001 and 29.04 ± 11.9 vs. 8.8 ± 3.4, P < 0.001 for CD3^−^CD16^bright^CD56^dim^ and CD3^−^CD16^dim^CD56^dim^, respectively). Furthermore, there were no significant differences in CD3^−^CD16^bright^CD56^bright^ and CD3^−^CD16^dim^CD56^bright^ subpopulations (1.11 ± 1.2 vs. 1.8 ± 2.9, P = 0.5 and 0.9 ± 1.5 vs. 1.6 ± 2.9, P = 0.4 respectively) for those with and without remission (Fig. 7b).

### Expression of CD244 and CD196 in different NK cell subpopulations and their relation to response

Dual expression of CD244 and CD196 on CD3^−^CD16^bright^CD56^bright^ was significantly higher on non-remitting compared with remitting ones (51.5 ± 12.9 vs. 31.7 ± 10.7, P < 0.001). Likewise CD3^−^CD16^bright^CD56^dim^ cells expressing only CD196 without co-expression of CD244 were significantly accumulated in non-remitting patients compared with those with remission (7.8 ± 4.4 vs. 3.4 ± 2.3, P = 0.017); conversely, absence of co-expression of CD244 and CD196 on CD3^−^CD16^bright^CD56^dim^ cells was significantly higher on remitting patients compared to non-remitting ones (3.3 ± 3.5 vs. 0.6 ± 0.7, P = 0.036). Cells with dim co-expression of both CD16 and CD56 and negative expression of CD244 and CD196 were significantly accumulated in remitting patients compared to those without remission (9.7 ± 7.9 vs. 4.0 ± 3.9, P = 0.05) (Fig. 7c). Other NK subpopulations showed no significant association with remission status.

We explored significant accumulation of CD3^−^CD16^+^CD56^+^ NK, CD3^−^CD16^+^CD56^+^CD244^−^CD196^−^ NK, and CD3^−^CD16^+^CD56^−^ NK cells in remitting patients compared to those without remission (39.9 ± 15.3 vs. 19.7 ± 15.4, P = 0.004, 6.2 ± 3.2 vs. 2.6 ± 2.8, P = 0.009, and 15.4 ± 6.8 vs. 8.3 ± 4.2, P = 0.009, respectively). No significant differences were observed in other subpopulations according to remission status (Fig. 7d).

### Association between immune cells and cytogenetics and risk groups

**Figure 8:**
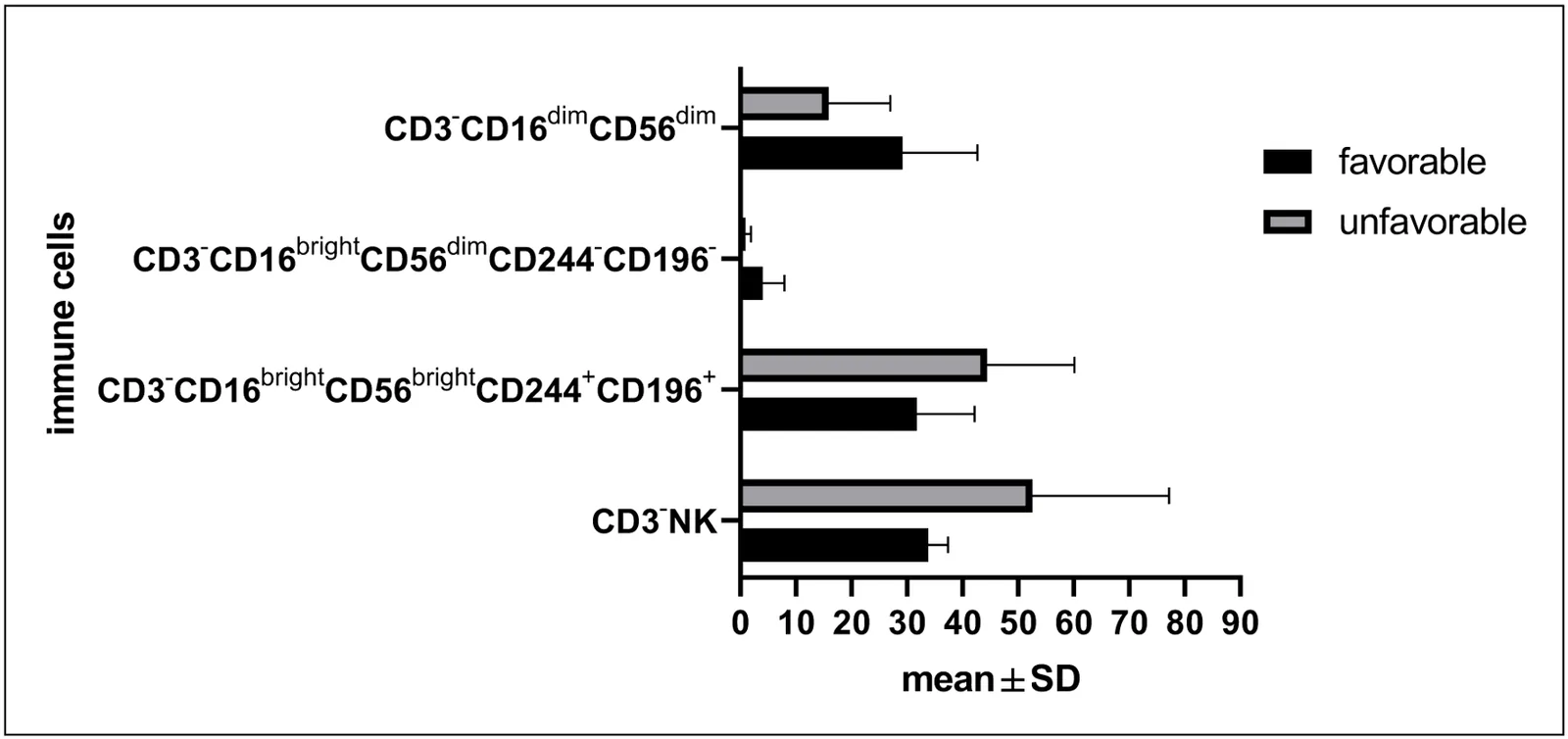
Differential expression of immune cells according to cytogenetics.

We did not detect any significant association between the current cytogenetics and different subpopulations of NKT including iNKT except CD3^+^CD16^+^CD56^+^CD244^+^CD196^−^ NKT cells which were accumulated in unfavorable cytogenetics (53.3 ± 24.9 vs. 73.3 ± 12.4, P = 0.022), but CD3^−^ NK cells were significantly increased in unfavorable cytogenetics compared to favorable ones (52.6 ± 24.6 vs. 33.8 ± 3.6 respectively, P = 0.014). CD3^−^CD16^bright^CD56^bright^CD244^+^CD196^+^ cells were accumulated in unfavorable cytogenetics (31.8 ± 10.4 vs. 44.4 ± 15.8, P = 0.024); however, the total CD3^−^CD16^dim^CD56^dim^ NK and CD3^−^CD16^bright^CD56^dim^CD244^−^CD196^−^ NK cells were significantly increased in favorable compared with unfavorable cytogenetics (29.2 ± 13.5 vs. 15.9 ± 11.1, P = 0.01 and 4.03 ± 3.9 vs. 0.9 ± 1.0, P = 0.008, respectively) (Fig. 8).

### Association between risk groups and immune cells

We detected significant accumulations of CD3^+^CD16^+^CD56^+^CD244^−^CD196^−^ NKT, CD3^−^CD16^bright^CD56^dim^, CD3^−^CD16^dim^CD56^dim^, and CD3^−^CD16^+^CD56^−^CD244^+^CD196^−^ in low and intermediate risk groups compared with the high-risk group, while the total CD3^−^ NK, CD3^−^CD16^bright^CD56^bright^ NK, and CD3^−^CD16^−^CD56^+^CD244^−^CD196^−^ NK cells were significantly accumulated in high-risk group compared with the other two groups (Table 7).

**Table 7:** Differential Expression of Immune Cells According to Risk Groups

Immune cells	Mean ± SD	P-value
CD3^+^CD16^+^CD56^+^CD244^−^CD196^−^ NKT		
Low risk	1.2 ± 0.9	0.05
Intermediate risk	0.42 ± 0.8	
High risk	0.42 ± 0.5	
CD3^−^ NK		
Low risk	32.8 ± 3.5	0.013
Intermediate risk	36.5 ± 9.3	
High risk	64.8 ± 25.0	
CD3^−^CD16^bright^CD56^dim^		
Low risk	12.4 ± 9.8	0.024
Intermediate risk	10.4 ± 6.2	
High risk	4.6 ± 2.7	
CD3^−^CD16^bright^CD56^bright^		
Low risk	31.8 ± 11.4	0.019
Intermediate risk	35.1 ± 13.5	
High risk	50.2 ± 14.0	
CD3^−^CD16^dim^CD56^dim^		
Low risk	30.4 ± 14.3	0.005
Intermediate risk	24.0 ± 12.7	
High risk	10.4 ± 3.7	
CD3^−^CD16^+^CD56^−^CD244^+^CD196^−^		
Low risk	43.9 ± 13.6	0.047
Intermediate risk	33.1 ± 12.3	
High risk	40.14 ± 18.6	
CD3^−^CD16^−^CD56^+^CD244^−^CD196^−^		
Low risk	9.5 ± 5.5	0.046
Intermediate risk	19.8 ± 11.6	
High risk	12.5 ± 6.7	

Data are expressed as mean ± SD and analyzed by Robust tests. P ≤ 0.05 is significant.

Furthermore, no significant correlations were observed between peripheral blasts and NKT subsets including iNKT cells; however, among different subgroups of NK cells, positive significant correlations were detected between blast cell frequency and CD3^−^CD16^dim^CD56^dim^CD244^+^CD196^−^ NK (r = 0.4, P = 0.041), and total CD3^−^CD16^−^CD56^+^NK (r = 0.4, P = 0.037), and significant negative correlations between blast frequency and the followings: CD3^−^CD16^dim^CD56^dim^CD244^−^CD196^+^ NK (r = −0.6, P = 0.001), CD3^−^CD16^dim^CD56^bright^CD244^+^CD196^−^ NK (r = ^−^0.6, P = 0.003), CD3^−^CD16^bright^CD56^dim^ NK (r = -0.5, P = 0.009), CD3^−^CD16^+^CD56^+^CD244^−^CD196^+^ NK (r = -0.63, P < 0.001), CD3^−^CD16^+^CD56^−^ NK (r = -0.6, P = 0.001), and CD3^−^CD16^−^CD56^+^CD244^−^CD196^+^ NK (r = −0.5, P = 0.012).

## Discussion

In normal population, NKT cells represent 5–10% of peripheral lymphocytes, while NK cells form about 1–20% and 3–28% of circulating lymphocytes in males and females, respectively [24, 25]. NK cells are actually capable of killing leukemic blasts through mechanisms based on receptor ligand interactions such as NKG2D and DNAM-1 receptors and NCRs (NKp30 and NKp46) which are reported important for targeting AML [26].

Studies showed that NK-mediated cytotoxicity was correlated with the expression of natural cytotoxicity receptors(NCRs), which are immunoglobulin like class 1 transmembrane molecules [27]. AML patients have been reported to show downregulated NCRs with subsequent suppressed NK cell functions and cytokine production in a mechanism by which AML blasts escape immune surveillance [28]. Previous reports suggested that better outcomes and prolonged remissions in AML could be associated with properly functioning NK cells [29].

The current results indicated significant accumulations of NK cells and different subpopulations including CD3^−^CD16^bright^CD56^bright^CD244^+^CD196^+^, 16^bright^CD56^dim^CD244^+^CD196^+^, CD3^−^CD16^bright^CD56^dim^CD244^−^CD196^+^, CD3^−^CD16^bright^CD56^dim^CD244^−^CD196^−^, CD3^−^CD16^dim^CD56^bright^CD244^+^CD196^+^cells, total CD3^−^CD16^dim^CD56^dim^cells, CD3^−^CD16^dim^CD56^dim^CD244^+^CD196^+^, CD3^−^CD16^dim^CD56^dim^CD244^−^CD196^−^, CD3^−^CD16^+^CD56^−^CD244^+^CD196^+^, CD3^−^CD16^+^CD56^−^CD244^+^CD196^−^, CD3^−^CD16^+^CD56^−^CD244^−^CD196^−^, total CD3^−^CD16^−^CD56^+^ cells, CD3^−^CD16^−^CD56^+^CD244^+^CD196^+^, and CD3^−^CD16^−^CD56^+^CD244^−^CD196^−^ cells in patients compared with controls.

Based on the surface expression of CD56, two distinct groups of NK cells were defined; 90% belonged to CD3^−^CD16^bright^CD56^dim^ group which are considered cytotoxic cells, and 10% belonged to CD3^−^CD56^bright^CD16^dim^ or CD3^−^CD56^+^CD16^−^ groups which exhibit more immunogenicity role by releasing cytokines [30]. Compared to the previous study, the most common subtype of NK cells in healthy controls was CD3^−^CD16^bright^CD56^dim^ representing ∼78% and the least subtype was CD3^−^CD56^bright^CD16^dim^ representing ∼2.5%, while CD3^−^CD16^dim^CD56^dim^ group represented the most common subtype in ∼65% of patients and the least one was CD3^−^CD56^bright^CD16^dim^ representing ∼3%. The average percentage of NK cells in AML patients was reported to be ≤ 11.7% [31] which was much lower than that displayed in the current study (mean percentage was 43.9%) and comparable to that reported for healthy controls (mean percentage was 11.8%).

Analysis of the phenotype and cytokine production has defined two major subsets of NKT cells: iNKT cells or type I NKT and diverse NKT cells or type II NKT. iNKT cells express antigen-specific TCR composed of semi-invariant α chain paired with a restricted repertoire of β chain, while type II NKT cells are restricted to CD1d and expressing polyclonal TCR repertoire comparable to the diverse TCR of normal CD4^+^ and CD8^+^ T cells [32]. Despite the semi-invariant TCR of iNKT cells, they recognize a diverse group of antigens, and iNKT cells can be activated during tumor immunosurveillance either directly by presentation of their self-lipids on CD1d positive tumor cells or indirectly by cross presentation of tumor lipids by antigen presenting cells.

CD1d is expressed on myelomonocytic leukemias, B-cell lineage malignancies, and multiple solid tumors including breast, prostatic, and renal cell cancers and CNS tumors [32]. Downregulation of CD1d by tumor cells inhibits iNKT-mediated cytotoxicity and results in disease progression and metastasis [33]. The average percentage of NKT cells in AML patients was reported to be 11.6% [31], which was much higher than that reported in our results (average percentage was 3.6%). iNKT that recognized glycolipid antigens presented by HLA class I has been examined in AML to display that levels lower than 0.2 cells/µL were associated with poor clinical outcomes [34]. Furthermore, one study indicated no significant difference in the percentage of NKT cells between AML patients and their healthy controls; however, it explored that decreased percentages of NKT cells were correlated with better outcomes in AML [35].

Conversely to the previously mentioned study, we detected a significantly elevated level of the total NKT cells in AML patients compared with controls and their increased levels were associated with remission but not related to clinical features including risk groups and cytogenetics. Our results were in alignment with Najera et al [34] where iNKT cells were significantly lower in patients compared with controls and increased levels were associated with better outcomes with no relation to clinical features. Another report denoted that the total NK cells and NKT cells were accumulated in non-remitting patients [31].

Previous reports investigated the frequency and functions of iNKT cells in the PB in hematologic malignancies and various solid tumors and observed reduced levels of these immune cells in patients compared with controls independent of tumor type [36–39], and these reduced levels correlated with worse outcomes in AML [34], and squamous cell carcinoma of head and neck [40]. On the other hand, other reports indicated that increased intra-tumoral or circulating levels of these cells were associated with good prognosis in hematologic malignancies, prostatic adenocarcinoma, colonic carcinoma, and neuroblastoma [41–43]. Comparable to these reports, we explored significantly lower levels of iNKT cells in AML patients compared with healthy volunteers, although increased levels were associated with good prognosis in AML patients. Terabe et al reported that targeting of iNKT cells is difficult due to high variability of iNKT cell frequencies and may be only effective in tumors with high iNKT cell frequency [32, 44].

CD56^bright^ NK cells have a higher expression of NKG2A, an inhibitory receptor, than CD56^dim^ NK cells which recognizes specific non-classical HLA molecule, HLA-E, which further transmits inhibitory signals to NK cells [45]. AML induces overexpression of NKG2A receptor on NK cells explaining the accumulation of CD56^bright^ NK in patients without remission [45, 46]. In the current results, CD56^dim^ NK cells were accumulated in those with remission, while CD56^bright^ NK cells were insignificantly increased in non-remitting patients.

CD16 is involved in antibody-dependent cellular cytotoxicity and mainly expressed on large granular cells including NK, NKT, and T cells. Dim expression of CD16 is detected in 15–20% of peripheral blood mononuclear neutrophils (PBMNs) and 5% of BM lymphocytes, while bright expression of CD16 is mainly detected in monocytes, macrophages, granulocytes, etc. CD16 is considered the powerful activating receptor to human NK inducing strong cytotoxicity and cytokine production [47, 48]. CD16 was downregulated in AML and correlated markedly with poor prognosis and decreased remission rate [49]. Our results came in alignment with the previous report where CD16^+^ expression irrespective of CD56 was associated with increased remission rate.

A recent study showed an evidence of moderate to high accumulation of CD16^+^CD56^−^ unconventional NK cells in 27% of AML patients. These NK cells displayed low expressions of activating receptors, NKG2A, NKp30, and NKp46. The increased level of these cells at time of diagnosis of AML was associated with adverse features and decreased remission rate and survival [50]. Contrary to that study, our results revealed a significant accumulation of these cells in heathy volunteers compared to newly diagnosed AML patients. This accumulation was associated with favorable prognostic features including lower risk groups and decreased percentage of peripheral blasts, and increased remission rate.

CD244 (2B4), which is a member of signaling lymphocyte activating molecule family of CD2-related receptors, is expressed by all NK cells. Two isoforms were described: one facilitates lysis of CD48-positive target tumor cells and the other does not. The dual function of 2B4 receptor is controlled partly by CD48 ligand density and the level of receptor expression [15]. Loss of 2B4 results in partial increase in NK lysis, while loss of its ligand greatly increases tumor lysis by NK cells [51]. CD244 appears to predominantly induce inhibitory signaling in tumor-associated immune cells, but the interplay of factors determining activating versus inhibitory signaling has not been fully clarified. In the current study, the expression level of 2B4 receptor showed specific pattern, where its expression when combined with CD196 resulted in accumulation of NK cells in AML patients compared with controls, while its positive expression with loss of CD196 resulted in accumulation of NK cells in controls. This effect was consistent across all types of NK cells. This pattern of expression was reversed in NKT cells. Furthermore, its expression was inconsistent with remission status and could be controlled with type of NK cells and CD196 receptor expression. A finding could be comparable with the previous reports; however, iNKT cells with positive CD244 and CD196 were accumulating in remission status while NKT cells only expressing CD244 were accumulated in non-remission state, suggesting that it exerted an inhibitory effect on NKT cells [52].

Tumor cells release signaling mediators such as cytokines that activate multiple pathways to establish a tumor-supportive microenvironment. In this context, chemokines mainly contribute to cancer progression by recruiting immune cells in a way that can benefit tumor growth. Specifically, interactions between chemokine receptors—such as CCR6 or CD196 expressed on NK and NKT cells—and their ligand CCL20, produced by tumor cells, have been linked to cancer development, tumor progression, and poorer prognosis, as reported in breast cancer [53], colorectal cancer [54], pancreatic cancer [55], lung cancer [56], and others [57]. In the current results, CCR6 expression was generally linked to higher accumulation of NKT cells in controls than in patients. Conversely, among NK cells, CCR6-expressing cells were more abundant in patients compared with controls. However, CD196^+^ NKT cells showed increased accumulation in non-remitting patients.

Moreover, CD196 positive expression on CD3^−^CD16^bright^CD56^dim^ NK cells was associated with no remission, while CD3^−^CD16^dim^CD56^dim^ NK and CD3^−^CD16^+^CD56^+^ NK cells losing CD196 expression were accumulated in remitting patients. In addition, CD196 expression was associated with poor prognostic features including high blast frequency and unfavorable cytogenetics. Our results came in line with the previously mentioned studies.

In a series of AML patients, the expression of CD244 on T cells was evident in relapsed cases than *de novo* ones, and its expression correlated with exhausted phenotype of T cells, decreased proliferation and expression of CD28, and increased expressions of immunosuppressive molecules such as PD1, TIM3, CTLA-4, and CD160 in cancer patients compared to healthy controls [58]. Other studies showed no correlations with PD1, proliferation, or cytokine production [59]. However, Epling-Burnette et al demonstrated that increased expression of CD244 was associated with decreased expression of activating molecules like CD28, CD62L [60]. Collectively CD244 in hematologic malignancies and cancer exhibited inhibitory effects on immune cells and immunosuppression with resistance to treatments and early relapse, in compliance with the previous theories. CD244 in the current results was associated with failure of response to treatments, unfavorable risk features, and cytogenetics. Knockdown of CD244 in AML resulted in impaired proliferation without comparable effect on normal hematopoietic stem cells [61]. Trials are ongoing to accurately evaluate the exact role and impact of CD244 and CD196 leukemia and their differential expressions on immune cells, taking into consideration their relation to the expression of CD16, CD56 on NK and NKT cells.

### Conclusion

Together, NK and NKT cells contribute not only to the pathogenesis of AML but also to the prognosis and overall survival of patients. In addition, the expression pattern of CD244 (2B4 receptor) and CD196 on NK and NKT cells was generally associated with remission status and prognostic features of AML patients.

## Supplementary Material

Suppl 1 Differential expression of NKT subpopulations including iNKT between AML patients and controls.

Suppl 2 Differential expression of CD244 and CD196 on CD3^−^CD16^bright^CD56^bright^ NK cells between patients and controls.

Suppl 3 Differential expression of CD244 and CD196 on CD3^−^CD16^bright^CD56^dim^ NK cells between patients and controls.

Suppl 4 Differential expression CD244 and CD196 on CD3^−^CD16^dim^CD56^bright^ and CD16^dim^CD56^dim^ subpopulations.

Suppl 5 Differential expression of CD3^−^CD16^+^CD56^−^ and CD3^−^CD16^−^CD56^+^ subpopulations between patients and controls.

## Data Availability

The authors declare that data supporting the findings of this study are available within the article.
