# Supplementary material for: Could the Immune Modulation of Invariant Natural Killer T Cell and the Expression of CD244 and CD196 on Natural Killer Cell Subtypes Impact the Treatment Outcomes of Acute Myeloid Leukemia Patients?
Source: J Hematol. 2026 Aug 31;15(4):198–213. doi: 10.14740/jh2209 (PMC13568744; doi:10.14740/jh2209)
Supplement: Supplementary file 1 [file jh-15-04-198-s001.docx]

**Suppl 1.** Differential expression of NKT subpopulations including iNKT between AML patients and controls.

| **NKT subgroups** | **group** | **Mean%± SD** | **p-value** |
| --- | --- | --- | --- |
| CD3^+^T cells | Patients | 61.4± 15.3 | <0.001 |
|  | controls | 74.1± 3.4 |  |
| CD3^+^CD16^+^CD56^+^ | patients | 3.6± 2.7 | 0.002 |
|  | controls | 1.7± 0.6 |  |
| CD3^+^CD16^+^CD56^+^CD244^+^CD196^+^iNKT | patients | 26.0± 14.2 | <0.001 |
|  | controls | 57.3± 17.8 |  |
| CD3^+^CD16^+^CD56^+^CD244^+^CD196^-^ | patients | 64.1± 21.4 | <0.001 |
|  | controls | 39.1± 19.4 |  |
| CD3^+^CD16^+^CD56^+^CD244^-^CD196^+^ | patients | 3.4± 4.7 | 0.014 |
|  | controls | 8.3± 7.8 |  |
| CD3^+^CD16^+^CD56^+^CD244^-^CD196^-^ | patients | 0.7± 0.8 | 0.09 |
|  | controls | 0.4± 0.5 |  |

Data expressed as mean± SD and analyzed by Mann-Whitney test, *p*≤0.05 is significant.
