# Supplementary material for: Could the Immune Modulation of Invariant Natural Killer T Cell and the Expression of CD244 and CD196 on Natural Killer Cell Subtypes Impact the Treatment Outcomes of Acute Myeloid Leukemia Patients?
Source: J Hematol. 2026 Aug 31;15(4):198–213. doi: 10.14740/jh2209 (PMC13568744; doi:10.14740/jh2209)
Supplement: Supplementary file 2 [file jh-15-04-198-s002.docx]

**Suppl 2.** Differential expression of CD244 and CD196 on CD3^-^CD16^bright^CD56^bright^ NK cells between patients and controls

| **CD3-CD16^bright^CD56^brifht^** | **Group** | **Mean%± SD** | **p-value** |
| --- | --- | --- | --- |
| CD3^-^CD16^bright^CD56^bright^ | patients | 1.3± 1.9 | 0.6 |
|  | controls | 1.1± 0.6 |  |
| CD3^-^CD16^bright^CD56^bright^ CD244^+^CD196^+^ | patients | 38.6± 14.8 | <0.001 |
|  | controls | 23.9± 9.9 |  |
| CD3^-^CD16^brigh^tCD56^bright^CD244^+^CD196^-^ | patients | 62.0± 14.9 | <0.001 |
|  | controls | 75.8± 9.8 |  |
| CD3^-^CD16^bright^CD56^bright^CD244^-^CD196^+^ | patients | 0.15± 0.4 | 0.7 |
|  | controls | 0.21± 0.4 |  |
| CD3^-^CD16^bright^CD56^bright^CD244^-^CD196^-^ | patients | 0.26± 0.6 | 0.2 |
|  | controls | 0.1± 0.2 |  |

Data expressed as mean%± SD and analyzed by Mann Whitney u-test, *p*≤0.05 is significant.
