# Supplementary material for: Could the Immune Modulation of Invariant Natural Killer T Cell and the Expression of CD244 and CD196 on Natural Killer Cell Subtypes Impact the Treatment Outcomes of Acute Myeloid Leukemia Patients?
Source: J Hematol. 2026 Aug 31;15(4):198–213. doi: 10.14740/jh2209 (PMC13568744; doi:10.14740/jh2209)
Supplement: Supplementary file 3 [file jh-15-04-198-s003.docx]

**Suppl 3.** Differential expression of CD244 and CD196 on CD3^-^CD16^bright^CD56^dim^ NK cells between patients and controls

| **CD3^-^CD16^bright^CD56^dim^ subpopulations** | **Group** | **Mean%± SD** | **p-value** |
| --- | --- | --- | --- |
| CD3^-^CD16^bright^CD56^dim^ | patients | 9.3± 7.5 | <0.001 |
|  | controls | 27.3± 3.9 |  |
| CD3^-^CD16^bright^CD56^dim^CD244^+^CD196^+^ | patients | 34.2± 11.1 | <0.001 |
|  | controls | 12.0± 3.1 |  |
| CD3^-^CD16^bright^CD56^dim^CD244^+^CD196^-^ | patients | 58.1± 11.01 | <0.001 |
|  | controls | 85.9± 3.3 |  |
| CD3^-^CD16^bright^CD56^dim^CD244^-^CD196^+^ | patients | 4.9± 3.8 | 0.031 |
|  | controls | 2.01± 3.1 |  |
| CD3^-^CD16^bright^CD56^dim^CD244^-^CD196^-^ | patients | 2.4± 3.1 | 0.001 |
|  | controls | 0.11± 0.2 |  |

Data expressed as mean%± SD, analyzed by Mann Whitney test, p≤0.05 is significant.
