# Supplementary material for: Could the Immune Modulation of Invariant Natural Killer T Cell and the Expression of CD244 and CD196 on Natural Killer Cell Subtypes Impact the Treatment Outcomes of Acute Myeloid Leukemia Patients?
Source: J Hematol. 2026 Aug 31;15(4):198–213. doi: 10.14740/jh2209 (PMC13568744; doi:10.14740/jh2209)
Supplement: Supplementary file 4 [file jh-15-04-198-s004.docx]

**Suppl 4.** Differential expression CD244 and CD196 on CD3^-^CD16^dim^CD56^bright^ and CD16^dim^CD56^dim^ subpopulations
