# Supplementary material for: Could the Immune Modulation of Invariant Natural Killer T Cell and the Expression of CD244 and CD196 on Natural Killer Cell Subtypes Impact the Treatment Outcomes of Acute Myeloid Leukemia Patients?
Source: J Hematol. 2026 Aug 31;15(4):198–213. doi: 10.14740/jh2209 (PMC13568744; doi:10.14740/jh2209)
Supplement: Supplementary file 5 [file jh-15-04-198-s005.docx]

**Suppl 5.** Differential expression of CD3^-^CD16^+^CD56^-^ and CD3^-^CD16^-^CD56^+^ subpopulations between patients and controls.

| **CD3^-^CD16^+^CD56^-^ subpopulations** | **Group** | **Mean%± SD** | ***p*-value** |
| --- | --- | --- | --- |
| CD3^-^CD16^+^CD56^-^ | patients | 12.9± 6.8 | <0.001 |
|  | controls | 22.3± 5.7 |  |
| CD3^-^CD16^+^CD56^-^CD244^+^CD196^+^ | patients | 20.2± 10.1 | <0.001 |
|  | controls | 6.7± 1.5 |  |
| CD3^-^CD16^+^CD56^-^CD244^-^CD196^+^ | patients | 24.3± 12.5 | <0.001 |
|  | controls | 63.9± 6.5 |  |
| CD3^-^CD16^+^CD56^-^CD244^+^CD196^-^ | patients | 39.02± 15.1 | 0.002 |
|  | controls | 27.6± 5.8 |  |
| CD3^-^CD16^+^CD56^-^CD244^-^CD196^-^ | patients | 17.2± 11.3 | <0.001 |
|  | controls | 1.9± 1.9 |  |
| **CD3^-^CD16^-^CD56^+^ subpopulations** | **Group** | **Mean%± SD** | ***p*-value** |
| CD3^-^CD16^-^CD56^+^ | patients | 18.9± 10.7 | <0.001 |
|  | controls | 3.6± 0.8 |  |
| CD3^-^CD16^-^CD56^+^CD244^+^CD196^+^ | patients | 25.7± 10.0 | 0.003 |
|  | controls | 15.0± 7.1 |  |
| CD3^-^CD16^-^CD56^+^CD244^-^CD196^+^ | patients | 17.7± 12.7 | <0.001 |
|  | controls | 37.7± 8.6 |  |
| CD3^-^CD16^-^CD56^+^CD244^+^CD196^-^ | patients | 42.6± 13.4 | 0.9 |
|  | controls | 42.1± 8.5 |  |
| CD3^-^CD16^-^CD56^+^CD244^-^CD196^-^ | patients | 14.0± 9.3 | <0.001 |
|  | controls | 5.3± 2.7 |  |

Data expressed as mean%± SD, analyzed by Mann Whitney test, p≤0.05 is significant.
